# Mapping evidence on charitable food assistance system’s compliance with safety and general hygiene requirements in Africa and the rest of the world: a systematic scoping review protocol

**DOI:** 10.1186/s13643-018-0907-2

**Published:** 2019-01-08

**Authors:** Sizwe Makhunga, Tivani Mashamba-Thompson, Khumbulani Hlongwana

**Affiliations:** Department of Public Health Medicine, School of Nursing and Public Health, University of KwaZulu-Natal, Durban, South Africa

**Keywords:** Food charitable organisations, Food redistribution/recovery practices, Food control management, Food safety and hygiene compliance, Charitable food assistance system

## Abstract

**Background:**

The charitable food assistance system has an influential role in the larger effort to curtail the problem of food insecurity globally. They often play a crucial role in meeting the food security needs of the poor, particularly resource-limited settings where food security is a challenge. The aim of our study is to compare evidence on the safety and general hygiene of charitable food assistance system in Africa and the rest of the world.

**Methods:**

This study will be conducted through a systematic scoping review. We will search for literature from the following electronic databases: African Index Medicus, PubMed, Google Scholar, EBSCOhost (MEDLINE with full text, Academic Search Complete, MEDLINE) and open access for unpublished theses and dissertations. Articles will also be searched through the ‘Cited by’ search as well as citations included in the reference lists of included articles. Websites, such as the World Health Organization (WHO) and the Departments of Health websites, will be searched for policies and guidelines for food charity practice. Studies will be identified by searching literature published in any language between 1967 and 2018. Then, following title screening, eligible studies will be exported to EndNote library where duplicates will be removed before the parallel screening of abstracts and full articles by two independent reviewers. Screening will be guided by the study eligibility criteria. Data from the included studies will be extracted, and the emerging themes will be analysed. The relationship of the emerging themes to the research question will be critically examined. The quality of the included studies will be determined by the use of Mixed Method Appraisal Tool (MMAT)—version 2011.

The search results will be presented through the use of the adapted PRISMA-P chart.

**Discussion:**

We hope to find relevant studies that highlight evidence of charitable organisations’ food safety and general hygiene practices, in order to effectively compare their performance to those of formal and informal food establishments. Findings of this study will be disseminated electronically, in print and through peer presentation, conferences and congresses on synergies between efforts to reduce food loss and waste and those practices of public health concern.

**Electronic supplementary material:**

The online version of this article (10.1186/s13643-018-0907-2) contains supplementary material, which is available to authorized users.

## Background

The contribution of poor food safety and general hygiene to foodborne illnesses continue to pose a public health threat in Africa, especially to vulnerable groups, such as children, the elderly and people with underlying diseases, including HIV/AIDS [1–3]. Each year, as many as 600 million, or almost 1 in 10 people in the world, fall ill after consuming contaminated food. Of these, 420,000 people die, including 125,000 children under the age of 5 years. This is despite the fact that children make up only 9% of the global population [1, 4]. While the burden of foodborne diseases is a public health concern globally, African and Southeast Asian regions have the highest incidence and death rates, inclusive of children under the age of 5 years [4]. In a recent study, Africa was estimated to have the highest burden of foodborne diseases, whereby more than 91 million people in the continent are estimated to fall ill and 137,000 die each year [3, 4].

Mishandling of food and the disregard for hygiene measures enable pathogens to come into contact with food and, in some cases, to survive and multiply in numbers sufficient to cause illness in consumers [5–7]. This applies to all food establishments (formal and informal), including the charitable food assistance system. In this study, we describe the charitable food assistance system to include food banks, food charitable organisations, pantries, community soup kitchens and emergency shelters. A critical question then becomes: to what degree the charitable food assistance system contribute to the above statistics?

This is a very critical question considering that although the charitable food assistance practice has existed in Africa and the rest of the world for a very long time through non-profit organisations (NPOs), non-governmental organisations (NGOs) and community-based organisations (CBOs), little is known about their performance in observing food safety and general hygiene requirements [8–10]. However, research has shown that the charitable food assistance system in Africa is still not comparable to that of the high-income countries [9, 10]. The research has further shown that the risk of foodborne diseases is most severe in low- and middle-income countries [1, 3, 4]. This is reported to be linked to one or more of the following practices: preparing food with unsafe water; poor safety and hygiene conditions in food production, preparation, processing, cooking or distribution; lower levels of literacy and education; and insufficient food safety and hygiene legislation or implementation of such legislation [4, 11–14].

The findings of this systematic scoping review will help us better understand how the charitable food assistance system in Africa and the rest of the world fare in ensuring food safety and in observing general hygiene requirements in one or more of the above-listed activities and practices, expose knowledge gaps and stimulate research to fill the gaps. Thus, the purpose of this systematic scoping review is to map the available evidence on the charitable food assistance system’s compliance with food safety and general hygiene requirements in Africa and the rest of the world. Our objectives are as follows:

- To highlight documented evidence on the charitable food assistance system in Africa and the rest of the world;
- To establish conditions under which the charitable food assistance system in Africa and the rest of the world operate;
- To compare the performance of the charitable food assistance system in Africa in observing food safety and general hygiene requirements to the charitable food assistance system in the rest of the world;
- To compare the performance of the charitable food assistance system in Africa in observing food safety and general hygiene requirements to that of other African formal and informal food establishments;
- To review the food control system applicable to the charitable food assistance system in Africa and the rest of the world.

## Methodology

### Study design

We will conduct a scoping review of peer-reviewed and grey literature on the available evidence of food charitable organisations’ compliance with food safety and general hygiene requirements in Africa and the rest of the world. This review will be guided by Arksey and O’Malley’s scoping review framework [15], which stipulates the following steps:

1. Identify the research question
2. Identifying relevant studies
3. Study selection
4. Charting the data
5. Collating, summarising and reporting the results

In addition, a quality assessment as recommended by Levac et al. [16] will be conducted in this study.

### Identifying the research question

The research question is: What is the evidence available on the charitable food assistance system’s compliance with food safety and general hygiene requirements in Africa and the rest of the world?

The sub-research questions are as follows:

- What is the state of food safety and general hygiene compliance in charitable food assistance system globally?
- What is the level of food safety and general hygiene in African charitable food assistance system compared to other formal and informal food establishments in Africa?
- What is the food control system applicable to the charitable food assistance system in Africa and the rest of the world?

### Eligibility criteria

The study will use the Population Concept Context (PCC) model [17] to determine the eligibility of the research question as illustrated in Additional file 1: Table S1.

### Identifying relevant studies

The review will include studies utilising qualitative, quantitative and mixed methods published in peer-reviewed journals as well as in grey literature addressing the research question. An electronic search of the following databases will be conducted: African Index Medicus, PubMed, Google Scholar, EBSCOhost (MEDLINE with full text, Academic Search Complete, MEDLINE) and open access for unpublished theses and dissertations. Articles will also be searched through the ‘Cited by’ search as well as citations included in the reference lists of included articles. Websites such as the World Health Organization (WHO) and governmental websites will be searched for policies and guidelines for food collection and redistribution. Studies will be identified by searching literature published in any language between 1967 and 2018. This is because the world’s first food recovery and redistribution practice in a form of a Food Bank was set up in 1967 in Phoenix, Arizona, in the USA by John van Hengel, and since then, thousands of food recovery and redistribution organisations have been set up all over the world [18, 19]. The search keywords will include food charitable organisations, food redistribution/recovery practices, food control management, food safety and hygiene compliance and charitable food assistance system. Search strategy will be piloted to check the appropriateness of the selected electronic databases and keywords (Additional file 2: Table S2).

### Study selection

The eligibility criteria will be developed to ensure specific information relating to the research question is included in the studies.

### Inclusion criteria

The review will include studies that present evidence of the following:

- Charitable food assistance system between 1967 and 2018;
- Charitable food assistance practices globally;
- Charitable food assistance practices rendering services for free (free food, volunteering etc.)
- Food safety and/or general hygiene requirements;
- Food hygiene and safety management
- Food control system

### Exclusion criteria

The review will exclude studies that present evidence of the following:

- Non-food charity practice;
- Charitable food assistance practice before 1967;
- Charitable food assistance practice rendering services for gain (monetary etc.)
- General hygiene and safety not associated with food;

The primary reviewer, the principal investigator (PI), will conduct a comprehensive search and screening of the study titles from the above-mentioned databases. All citations after the searches will be exported to the EndNote library, and all duplicates will be removed before embarking on abstract and full article screening. Two reviewers will conduct abstract followed by full article screening of the selected studies independently with guidance from the eligibility criteria.

To enhance the article search procedure, the primary reviewer will consult a subject librarian at the UKZN library services to seek help with retrieving and finding articles to be included in the full-article screening. The subject librarian for Public Health in the University of KwaZulu-Natal will also be approached for help with full article studies that are not on open access.

The screening results will be reported by the use of the adapted PRISMA chart (Additional file 3: Figure S1).

### Charting of data

A data charting tool will be developed using google forms to extract relevant information from each screened and selected study. It will be piloted. Data charting table (Additional file 4: Table S3) will be used to extract background information and process the information from each utilised study. The variables and themes included will be continually updated to effectively answer the research question.

### Collating, summarising and reporting the results

A narrative account of the data extracted from the included studies will be analysed using the thematic content analysis. Data will be extracted around the following themes: motives for charitable food assistance practice, benefits, challenges and barriers of charitable food assistance system, food safety and general hygiene requirements, vulnerability of donated ‘surplus food’, food hygiene and safety management, vulnerability of beneficiaries of donated ‘surplus food’ and applicable food control system. Emerging themes will also be extracted. The resulting themes will be analysed and their relationship (significance and relevance) to the research question critically examined. Reviewers will also analyse the implication of the findings in relation to the aim of the study as well as to future research and evidential framework for policy and practice in low- and medium-income settings. Recommended general food safety and general hygiene requirements and guidelines that are applicable to the charitable food assistance system will be drawn from the study evidence.

### Quality appraisal

The quality of the included primary studies will be appraised using the Mixed Method Appraisal Tool (MMAT)—version 2011 [20, 21]. For appraising a qualitative study, we will use section 1 of the MMAT; for a quantitative study, we will use section 2 for randomised controlled, section 3 for non-randomised, and section 4 for descriptive studies. For a mixed methods study, we will use section 1 for appraising the qualitative component, the appropriate section for the quantitative component (2, 3 or 4) and section 5 for the mixed methods component. The tool will be used to examine the appropriateness of study aims, the context relevance, theoretical inferences to answer research questions, author’s discussions and conclusions. The overall quality for each of the studies selected will be calculated following the MMAT guidelines (score = number of criteria met/4) and then presented using the following descriptors:

- Low quality (1–25%), where minimal criteria are met
- Average (26–50%)
- Above average (51–75%)
- High quality (876–100%), where all criteria are met.

For mixed methods studies, the principle is that the overall quality of a combination cannot be more than the quality of its weakest component [20, 21]. Consequently, the overall quality score will be the lowest score of the study components (qualitative or quantitative).

## Discussion

Correct food safety and hygiene practices help maximise the recovery and collection of surplus food from the entire agro-food supply chain. The supply chain consists of producers, distributors and retailers. The surplus food may be as a result of various reasons, including surplus production, which is food that does not meet specific aesthetic standards. Sometimes, it is due to incorrectly labelled products, which are unfit for sale but safe for human consumption. More often than not, it is due to foods with damaged packaging, food too close to its ‘use-by-date’, food leftovers and surplus from catering and canteen services. To date, most of the research into food recovery and redistribution in Africa and the rest of the world has focused on their function in relation to waste management and social problems of hunger and food insecurity [18, 22–26]. However, a recent systematic review by Neff et al. [27] concluded that there is a need for higher evidence research around the synergies between efforts to reduce food loss and waste and those promoting public health. This study will map the literature around to expose knowledge gaps from which the higher evidence research can be built on.

Results from the proposed study would be valuable for use in ensuring that food safety and general hygiene practices are addressed from production to consumption [28], as well as the development of appropriate hygiene and safety intervention strategies for charitable food assistance system in Africa and the rest of the world. This comprehensive and integrated approach is known as ‘farm to fork’, ‘stable to table’ or ‘boat to throat’ [28]. It implies that the responsibility of providing safe food to the consumer is shared by all stakeholders along the chain [28]. Findings of this study will be disseminated electronically, in print and through peer presentation, conferences and congresses advocating synergies between efforts to reduce food loss and waste and those promoting public health.

## Additional files

Additional file 1: **Table S1.** PCC model. (DOCX 13 kb)

Additional file 2: **Table S2.** Pilot database search results. (DOCX 13 kb)

Additional file 3: **Figure S1.** Flowchart of the search and selection process of studies on charitable food assistance system’s compliance with safety and general hygiene requirements in Africa and the rest of the world. (DOCX 49 kb)

Additional file 4: **Table S3.** Data charting table. (DOCX 14 kb)

## Abbreviations

CBOs: Community-based organisations
HIV/AIDS: Human immunodeficiency virus/acquired immune deficiency syndrome
MMAT: Mixed Method Appraisal Tool
NGOs: Non-governmental organisations
NPOs: Non-profit organisations
PCC: Population Concept Context
PI: Principal investigator
PRISMA: Preferred Reporting Items for Systematic Reviews and Meta-Analyses
UKZN: University of KwaZulu-Natal
WHO: World Health Organization
