# Supplementary material for: Mapping evidence on charitable food assistance system’s compliance with safety and general hygiene requirements in Africa and the rest of the world: a systematic scoping review protocol
Source: Syst Rev. 2019 Jan 8;8:10. doi: 10.1186/s13643-018-0907-2 (PMC6323661; doi:10.1186/s13643-018-0907-2)
Supplement: Supplementary file 1 — Table S1. PCC model. (DOCX 13 kb) [file 13643_2018_907_MOESM1_ESM.docx]

**Table S1** PCC Model

__________________________________________________________________________

**Criteria Determinant**

__________________________________________________________________________

Population Charitable food assistance system in Africa and the rest of the world.

Concept/Intervention Food safety and general hygiene compliance

Context Africa and the rest of the world

___________________________________________________________________________
