# Supplementary material for: Mapping evidence on charitable food assistance system’s compliance with safety and general hygiene requirements in Africa and the rest of the world: a systematic scoping review protocol
Source: Syst Rev. 2019 Jan 8;8:10. doi: 10.1186/s13643-018-0907-2 (PMC6323661; doi:10.1186/s13643-018-0907-2)
Supplement: Supplementary file 2 — Table S2. Pilot database search results. (DOCX 13 kb) [file 13643_2018_907_MOESM2_ESM.docx]

**Table S2.** Pilot database search results

___________________________________________________________________________

Criteria Date of Searched Search Engine No. Retrieved

___________________________________________________________________________

((("food"[MeSH Terms] OR 2017/01/18 PubMed 8605

"food"[All Fields])

AND ("safety"[MeSH Terms] OR

"safety"[All Fields]))

OR ("hygiene"[MeSH Terms] OR

"hygiene"[All Fields]))

AND ("africa"[MeSH Terms] OR

"africa"[All Fields])

___________________________________________________________________________
