# Supplementary material for: Mapping evidence on charitable food assistance system’s compliance with safety and general hygiene requirements in Africa and the rest of the world: a systematic scoping review protocol
Source: Syst Rev. 2019 Jan 8;8:10. doi: 10.1186/s13643-018-0907-2 (PMC6323661; doi:10.1186/s13643-018-0907-2)
Supplement: Supplementary file 3 — Figure S1. Flowchart of the search and selection process of studies on charitable food assistance system’s compliance with safety and general hygiene requirements in Africa and the rest of the world. (DOCX 49 kb) [file 13643_2018_907_MOESM3_ESM.docx]

Identification

Additional records through other sources

(n=61)

Records identified through database searching

(n=713)

Records after duplicates removed

(n=579)

(

Screening

Records excluded

(n=10)

Records screened by abstracts

(n=48)

Studies included for data content analyses

(n=23)

Studies included for quality assessment

(n=23)

Eligibility

Full-text articles assessed for eligibility

(n=38)

Full-text articles excluded with reasons (n = 15):

- Waste management strategy (n=4)
- Food loss prevention studies (n=4)
- Social impacts studies (n=2)
- Food flow in FCOs (n=2)
- Food retail charity practice (n=2)
- Korean study (n=1) – no translator

Included

**Figure S1** Flow chart of the search and selection process of studies on charitable food assistance system’s compliance with safety and general hygiene requirements in Africa and the rest of the world.
