# Supplementary material for: Mapping evidence on charitable food assistance system’s compliance with safety and general hygiene requirements in Africa and the rest of the world: a systematic scoping review protocol
Source: Syst Rev. 2019 Jan 8;8:10. doi: 10.1186/s13643-018-0907-2 (PMC6323661; doi:10.1186/s13643-018-0907-2)
Supplement: Supplementary file 4 — Table S3. Data charting table. (DOCX 14 kb) [file 13643_2018_907_MOESM4_ESM.docx]

**Table S3** Data Charting Table

__________________________________________________________________

Author and date

Journal full reference

Aims/research questions

Recruitment context/study population

Geographic setting

Study design

Data collection methods used

Data analysis employed

Interventions (Food safety and general hygiene requirements)

Disease burden/vulnerability of beneficiary population

Priorities (public health/social vs environmental/waste management)

Different motives for charitable food assistance system

Benefits, challenges and barriers of charitable food assistance system

Vulnerability of donated “surplus food”

Charitable food assistance system’s food control

Conclusions

___________________________________________________________________
